# Comparison of Xpert MTB/RIF with Other Nucleic Acid Technologies for Diagnosing Pulmonary Tuberculosis in a High HIV Prevalence Setting: A Prospective Study

**DOI:** 10.1371/journal.pmed.1001061

**Published:** 2011-07-26

**Authors:** Lesley E. Scott, Kerrigan McCarthy, Natasha Gous, Matilda Nduna, Annelies Van Rie, Ian Sanne, Willem F. Venter, Adrian Duse, Wendy Stevens

**Affiliations:** 1 Department of Molecular Medicine and Hematology, University of the Witwatersrand, Johannesburg, South Africa; 2 Wits Reproductive Health and HIV Institute, University of the Witwatersrand, Johannesburg, South Africa; 3 National Health Laboratory Service, Johannesburg, South Africa; 4 Department of Epidemiology, University of North Carolina at Chapel Hill, Chapel Hill, North Carolina, United States of America; 5 Clinical HIV Research Unit, Department of Medicine, University of the Witwatersrand, and Right to Care, Johannesburg, South Africa; 6 Department of Clinical Microbiology and Infectious Diseases, University of the Witwatersrand, Johannesburg, South Africa; McGill University, Canada

## Abstract

**Background:**

The Xpert MTB/RIF (Cepheid) non-laboratory-based molecular assay has potential to improve the diagnosis of tuberculosis (TB), especially in HIV-infected populations, through increased sensitivity, reduced turnaround time (2 h), and immediate identification of rifampicin (RIF) resistance. In a prospective clinical validation study we compared the performance of Xpert MTB/RIF, MTBDRplus (Hain Lifescience), LightCycler Mycobacterium Detection (LCTB) (Roche), with acid fast bacilli (AFB) smear microscopy and liquid culture on a single sputum specimen.

**Methods and Findings:**

Consecutive adults with suspected TB attending a primary health care clinic in Johannesburg, South Africa, were prospectively enrolled and evaluated for TB according to the guidelines of the National TB Control Programme, including assessment for smear-negative TB by chest X-ray, clinical evaluation, and HIV testing. A single sputum sample underwent routine decontamination, AFB smear microscopy, liquid culture, and phenotypic drug susceptibility testing. Residual sample was batched for molecular testing. For the 311 participants, the HIV prevalence was 70% (*n* = 215), with 120 (38.5%) culture-positive TB cases. Compared to liquid culture, the sensitivities of all the test methodologies, determined with a limited and potentially underpowered sample size (*n* = 177), were 59% (47%–71%) for smear microscopy, 76% (64%–85%) for MTBDRplus, 76% (64%–85%) for LCTB, and 86% (76%–93%) for Xpert MTB/RIF, with specificities all >97%. Among HIV+ individuals, the sensitivity of the Xpert MTB/RIF test was 84% (69%–93%), while the other molecular tests had sensitivities reduced by 6%. TB detection among smear-negative, culture-positive samples was 28% (5/18) for MTBDRplus, 22% (4/18) for LCTB, and 61% (11/18) for Xpert MTB/RIF. A few (*n* = 5) RIF-resistant cases were detected using the phenotypic drug susceptibility testing methodology. Xpert MTB/RIF detected four of these five cases (fifth case not tested) and two additional phenotypically sensitive cases.

**Conclusions:**

The Xpert MTB/RIF test has superior performance for rapid diagnosis of *Mycobacterium tuberculosis* over existing AFB smear microscopy and other molecular methodologies in an HIV- and TB-endemic region. Its place in the clinical diagnostic algorithm in national health programs needs exploration.

*Please see later in the article for the Editors' Summary*

## Abstract 2

In this prospective, real-world cohort study nested within a national screening program for tuberculosis, Lesley Scott and colleagues compare the performance of Xpert MTB/RIF on a single sputum sample with different TB sputum detection technologies.

## Editors' Summary

**Background:**

Tuberculosis (TB)—a contagious bacterial infection that mainly affects the lungs—is a global public health problem. In 2009, 9.4 million people developed TB, and 1.7 million people died from the disease; a quarter of these deaths were in HIV-positive individuals. People who are infected with HIV, the virus that causes AIDS, are particularly susceptible to TB because of their weakened immune system. Consequently, TB is a leading cause of illness and death among people living with HIV. TB is caused by *Mycobacterium tuberculosis*, which is spread in airborne droplets when people with the disease cough or sneeze. Its characteristic symptoms are a persistent cough, night sweats, and weight loss. Diagnostic tests for TB include sputum smear analysis (the microscopic examination of mucus brought up from the lungs by coughing for the presence of *M. tuberculosis*) and mycobacterial liquid culture (in which bacteriologists try to grow *M. tuberculosis* from sputum samples and test its drug sensitivity). TB can usually be cured by taking several powerful drugs daily for at least six months.

**Why Was This Study Done?:**

Mycobacterial culture is a sensitive but slow way to diagnose TB. To halt the disease's spread, it is essential that TB—particularly TB that is resistant to several treatment drugs (multidrug-resistant, or MDR, TB)—is diagnosed quickly. Recently, several nucleic acid amplification technology (NAAT) tests have been developed that rapidly detect *M. tuberculosis* DNA in patient samples and look for DNA changes that make *M. tuberculosis* drug-resistant. In December 2010, the World Health Organization (WHO) endorsed Xpert MTB/RIF—an automated DNA test that detects *M. tuberculosis* and rifampicin resistance (an indicator of MDR TB) within two hours—for the investigation of patients who might have TB, especially in regions where MDR TB and HIV infection are common. TB diagnosis in HIV-positive people can be difficult because they are more likely to have smear-negative TB than HIV-negative individuals. In this prospective study, the researchers compare the performance of Xpert MTB/RIF on a single sputum sample with that of smear microscopy, liquid culture, and two other NAAT tests (MTBDRplus and LightCycler Mycobacterium Detection) in adults who might have TB in Johannesburg (South Africa), a region where many adults are HIV-positive.

**What Did the Researchers Do and Find?:**

The researchers evaluated adults with potential TB attending a primary health care clinic for TB according to national guidelines and determined their HIV status. A sputum sample from 311 participants underwent smear microscopy, liquid culture, and drug susceptibility testing; 177 samples were also tested for TB using NAAT tests. They found that 70% of the participants were HIV-positive and 38.5% had culture-positive TB. Compared to liquid culture, smear microscopy, MTBDRplus, LightCycler Mycobacterium Detection, and Xpert MTB/RIF had sensitivities of 59%, 76%, 76%, and 86%, respectively. That is, assuming that liquid culture detected everyone with TB, Xpert MTB/RIF detected 86% of the cases. The specificity of all the tests compared to liquid culture was greater than 97%. That is, they all had a low false-positive rate. Among people who were HIV-positive, the sensitivity of Xpert MTB/RIF was 84%; the sensitivities of the other NAAT tests were 70%. Moreover, Xpert MTB/RIF detected TB in 61% of smear-negative, culture-positive samples, whereas the other NAATs detected TB in only about a quarter of these samples. Finally, although some TB cases were identified as drug-resistant by one test but drug-sensitive by another, the small number of drug-resistant cases means no firm conclusions can be made about the accuracy of drug resistance determination by the various tests.

**What Do These Findings Mean?:**

Although these findings are likely to be affected by the study's small size, they suggest that Xpert MTB/RIF may provide a more accurate rapid diagnosis of TB than smear microscopy and other currently available NAAT tests in regions where HIV and TB are endemic (i.e., always present). Indeed, the reported accuracy of Xpert MTB/RIF for TB diagnosis—85% sensitivity and 97% specificity—has the potential to save more than 400,000 lives per year. Taken together with the results of other recent studies (including an accompanying article by Lawn et al. that investigates the use of Xpert MTB/RIF for screening for HIV-associated TB and rifampicin resistance), these findings support the WHO recommendation that Xpert MTB/RIF, rather than smear microscopy, should be the initial test in HIV-infected individuals who might have TB.

**Additional Information:**

Please access these Web sites via the online version of this summary at http://dx.doi.org/10.1371/journal.pmed.1001061.

- This study is further discussed in a *PLoS Medicine* Perspective by Carlton Evans; a related *PLoS Medicine* Research Article by Lawn et al. is also available
- WHO provides information (in several languages) on all aspects of tuberculosis, including general information on tuberculosis diagnostics and specific information on the Xpert MTB/RIF test; further information about WHO's endorsement of Xpert MTB/RIF is included in a recent Strategic and Technical Advisory Group for Tuberculosis report
- WHO also provides information about tuberculosis and HIV
- The US National Institute of Allergy and Infectious Diseases has detailed information on tuberculosis and HIV/AIDS
- The US Centers for Disease Control and Prevention also has information about tuberculosis, including information on the diagnosis of and on tuberculosis and HIV co-infection
- Information is available from Avert, an international AIDS charity on many aspects of HIV/AIDS, including information on HIV-related tuberculosis (in English and Spanish)

## Introduction

The tuberculosis (TB) and HIV epidemic in sub-Saharan Africa continues to pose enormous challenges to public health. South Africa alone has 1 million people currently receiving HIV antiretroviral treatment [1],[2], the TB incidence is 941 per 100,000 individuals [3], and 9,070 cases of multidrug-resistant (MDR) TB were reported in 2009 [4]. A recent post-mortem study from KwaZulu-Natal observed that TB is still the leading cause of death in HIV+ individuals [5], suggesting that the diagnosis of TB is made too late to avert mortality. Early diagnosis and management of TB is also critical to reduce TB transmission in communities and health care facilities. In 2009, 3 million smears and 740,000 cultures were performed in South Africa public sector health care facilities (excluding the KwaZulu Natal province) [6],[7]. In recent years, the South African National Health Laboratory Service scaled up its infrastructure to 249 sites for smear microscopy and 16 laboratories for Mycobacteria Growth Indicator Tube (MGIT) culture. While culture remains the most sensitive method for confirmation of TB, the prolonged turnaround time, biosafety requirements, and laboratory operational requirements [8] limit its contribution to clinical decision making [9]. In 2009, the World Health Organization (WHO) approved the MTBDRplus assay (Hain Lifescience) for use in smear-positive specimens and *Mycobacterium tuberculosis* (*M.tb*) isolates [10]. To hasten diagnosis of drug-resistant TB, 20 National Health Laboratory Service laboratories were earmarked for implementation of the MTBDRplus assay in South Africa in 2010. The assay is limited in its application because laboratory infrastructure must accommodate PCR technology, the assay is not approved for use in smear-negative cases, and in high-throughput laboratories, it can take up to 7 d from sample receipt to result reporting [6]. All these factors limit its potential to contribute to the control of drug-resistant TB.

The diagnostic development pipeline for both high-throughput and point-of-care laboratories has seen rapid innovations in the last decade [11],[12] through WHO, Stop TB Partnership, and Foundation for Innovative New Diagnostics partnerships [13],[14]. The most recently WHO-endorsed [15] diagnostic tool, the Xpert MTB/RIF (Cepheid) assay, has been reported in a multi-country study to have sensitivities of 98.2% among smear-positive, culture-positive patients and 72.5% among smear-negative, culture-positive patients on a single direct Xpert MTB/RIF test compared to three smears and four culture results [16]. Two sites from South Africa (Durban and Cape Town) with average HIV infection rates of 73% were included in this multi-center study. The sensitivity of the Xpert MTB/RIF assay (three tests performed per participant) among smear-negative, culture-positive individuals compared to standard testing (three smears and four culture results per participant) was 87% (95% CI 62%–96%) for samples from Durban and 90% (95% CI 79%–96%) for samples from Cape Town. Other studies have also recently reported the performance and clinical role of the Xpert MTB/RIF test for detecting TB in extrapulmonary specimens, with sensitivities of 69% to 85.7% for tissue specimens and up to 100% in urine and stool specimens [17],[18].

Our study aims to (1) further assess the performance of a single-sputum Xpert MTB/RIF test against culture-confirmed and clinically defined cases of TB in a cohort of adults being investigated for TB with high prevalence of HIV infection from South Africa (Johannesburg region) and (2) to compare this nucleic acid amplification technology (NAAT) to two existing molecular TB assays, the LightCycler Mycobacterium Detection (LCTB) assay (Roche) and the MTBDRplus assay (Hain Lifescience), for use directly on sputum.

## Methods

### Ethics Approval

The study was approved by the University of the Witwatersrand Human Ethics Review Committee (M070826).

### Study Design for Investigation of Potential TB Patients and Data Management

This prospective study investigated consecutive adults presenting with suspected pulmonary TB to a primary health care clinic in Johannesburg, South Africa, over a 9-mo period between 3 August 2009 and 28 May 2010. Individuals were eligible if they were ≥18 y of age and presented with a cough of ≥2 wk duration, with or without fever, night sweats, loss of weight, chest pain, and signs of extrapulmonary involvement (such as lymph nodes, pleural effusions, or abdominal TB), independent of a history of TB treatment and acceptance of HIV testing. Persons were excluded if they were not able to produce sputum, had symptoms only of extrapulmonary TB, were already on TB treatment, or required hospital admission.

TB and HIV diagnosis and management were performed according to South African guidelines [1],[19]. As part of routine care, participants on first presentation were asked to provide two sputum specimens for smear microscopy. On return (within 1 wk) for results, participants were invited to participate, and written informed consent was obtained by the study nurse. At this visit, all participants were asked to provide a third sputum specimen for routine smear and culture, and investigational tests. Smear-positive patients were started on TB treatment. AFB-smear-negative patients underwent chest radiography and were prescribed amoxicillin. One week later, response to antibiotic therapy was evaluated, the chest X-ray was read, and the case was assessed by the study physician. Smear-negative participants with no response to antibiotics and chest X-ray findings compatible with TB were initiated on TB treatment. Participants without these criteria were deemed not to have TB. When in doubt, participants were referred to a tertiary center for further investigation. For all participants, data were collected on history of TB, HIV status, most recent CD4 count, antiretroviral therapy, weight, and oral temperature at baseline, and a follow-up visit was conducted approximately 60 d after enrolment. The third sputum sample underwent routine and immediate *N*-acetyl-L-cysteine-sodium hydroxide (NALC)–NaOH decontamination for AFB smear and MGIT culturing. Residual processed specimen was stored at −70°C, batched, and later used for the three NAAT tests. The NAAT tests were performed by a scientist, blinded to smear, culture, and clinical evaluations in an off-site laboratory. All culture-positive specimens underwent routine MTBDRplus testing for rifampicin (RIF) and isoniazid (INH) resistance, and where one or other of these was found present, phenotypic drug sensitivity testing (DST) was performed. These routine smear and culture DST results were reported to clinicians and used for patient management and clinical decision making. The sensitivity, specificity, and positive and negative predictive values for the NAAT tests compared to MGIT culture and clinical case definition were calculated from the results generated from this single processed sputum sample.

Patient data were recorded using a standardized case report form, entered periodically into MS Access and exported into STATA 10 (StataCorp) for analysis. Characteristics between groups were compared using chi-square and *t*-tests as appropriate. Sensitivity, specificity, and positive and negative predictive values were calculated using either MGIT culture (excluding contaminated cultures and non-tuberculous mycobacteria [NTM]) or any TB (definite, probable, and possible TB) as a gold standard. NAAT test performance was established for those specimens where sufficient sample allowed all tests to be done on each specimen. Analysis was stratified by HIV status and smear microscopy.

### Definitions for TB Case Classification

Participating individuals were classified as “definite TB” if sputum culture yielded *M.tb* (with or without positive smears); “probable TB” if *M.tb* culture was negative/contaminated and at least one smear was positive for AFB; “possible TB” if smear was negative for AFB, *M.tb* culture was negative or contaminated, but the patient had TB-compatible chest X-ray and any documented weight gain in response to TB treatment; and “no TB” if smear was negative for AFB, *M.tb* culture was negative or contaminated, symptoms resolved without TB treatment, or if the culture grew NTM. Individuals who were smear-negative, had a culture that was negative or contaminated, had a chest X-ray suggestive of TB, and were initiated on TB treatment, but in whom weight gain was not documented, were classified as “indeterminate TB.” Participants who were not started on TB treatment and were lost to follow-up or died were also classified as indeterminate TB. The clinical classification of TB status was performed blinded to the NAAT results.

### Laboratory Methods

The single sputum sample was processed and analyzed using standard operating procedures in an accredited biosafety level 3 laboratory. Following decontamination using NaOH (1%)–NALC [20], the specimen was centrifuged and resuspended in approximately 2 ml of phosphate buffer (pH 6.8) to ensure maximum recovery of bacteria and easy homogenization before aliquots were removed for testing methodologies. The reconstituted pellet was used fresh for smear microscopy (∼50 µl) and culture (0.5 ml), and the residual sample was stored at −70°C for NAAT processing. The MTBDRplus and the LCTB assays were the first NAATs to be performed in batches of 12 per day (extraction protocols performed on day 1 followed by amplification the following day). Once the Xpert MTB/RIF became available (June 2009), 4–5 residual frozen (−70°C) specimens stored after completion of the MTBDRplus and LCTB assays were tested daily. Use of residual pellet for Xpert MTB/RIF (0.5 ml), MTBDRplus (0.5 ml), and LCTB (0.1 ml) depended on availability of residual sample after smear and culture. Any specimen yielding an invalid NAAT result was re-tested if there was sufficient residual material. This latter result was used in the sensitivity and specificity calculations.

The sputum smear was stained using standard auramine reagent and 100 high-power fields examined using a fluorescent microscope (Olympus CX31 with LED attachment, Wirsam). Culture was performed using MGIT containing modified Middlebrook 7H9 broth base, supplemented with MGIT Growth Supplement and PANTA (BD) and incubated at 37°C up to 42 d in a BACTEC cabinet (Becton Dickinson). Positive cultures were subjected to Ziehl-Neelsen staining to confirm the presence of AFB, and to routine MTBDRplus assay to confirm identity as *M.tb* and establish INH and RIF susceptibility profiles. Routine phenotypic MGIT DST was performed when MTBDRplus assay detected genotypic resistance. All cultures were preserved and stored. At completion of the study MGIT DST was performed as per manufacturer's instructions on additional isolates when this had not been done before.

All NAAT methods were performed according to the manufacturer's instructions and are detailed below. The LCTB assay is a real-time PCR assay, with bacterial nucleic acid extracted using the COBAS Amplicor Respiratory Specimen Preparation kit (Roche Diagnostics) by adding a wash and lysis solution to the pellet followed by 45 min incubation at 60°C and addition of a neutralization buffer before the PCR step. PCR is performed using the LCTB amplification kit (Roche Diagnostics) designed to amplify a 200-bp fragment of the 16s rRNA gene containing the hypervariable region A using fluorescent resonance energy transfer hybridization probes designed for the LightCycler instrument (Roche Diagnostics). Melting curve analysis is performed for species differentiation (positive control, 59±1.5°C; negative control not defined; *M.tb*, 55.9±1.5°C; *M. kansasii*, 59±1.5°C; *M. avium*, 47.5±1.5°C).

The MTBDRplus assay in this study was performed directly on sputum (irrespective of smear result) and routinely on positive cultures. In this assay, bacterial nucleic acid extraction is performed by heat followed by sonication. The PCR is a multiplex amplification using biotinylated primers, followed by reverse hybridization onto nitrocellulose strips. A strip contains 17 probes, including five sample and hybridization controls [21]. The targets amplified are (1) the core region of the *rpoB* gene, positions 505–533, analyzed for RIF resistance based on eight wild-type probes and four mutant probes (D516V, H526Y, H526D, and S531L), (2) the *katG* gene, analyzed for high-level INH resistance based on the wild-type S315 and two mutants (AGC to ACC and AGC to ACA, both producing S315T mutations), and (3) the *inhA* gene, analyzed for low-level INH resistance based on the wild-type 1 probe spanning positions 9–22 and wild-type 2 probe spanning positions 1–12, as well as four mutation probes (C15T,1A6G, T8C, and T8A) [21]. After several washes and chromogenic substrate reaction, the bound probes are visually inspected for the presence or absence of control, wild-type, and mutant bands. Omission of a wild-type band or the appearance of a mutant band in the resistance-determining region of a gene indicates the existence of a resistant strain.

The Xpert MTB/RIF assay is a hemi-nested real-time PCR method that amplifies the 81-bp region of the RIF-resistance-determining region of the *rpoB* gene, positions 507–533. A sample reagent buffer containing NaOH and isopropanol is added in a 2∶1 ratio to the processed sputum ensuring a final volume of at least 2 ml. After 15 min of incubation with intermittent hand mixing, 2 ml of the liquefied inactivated sample is added to the cartridge that contains the wash buffer, reagents for lyophilized DNA extraction and PCR amplification, and fluorescent detection probes (five for the *rpoB* gene and one for an internal control, *Bacillus globigii* spores). After the cartridge is placed in the instrument module, the automated processes include the following: specimen filtering, sonication to lyse the bacilli and internal control spores, released DNA collection and combination with the PCR reagents, amplification, target detection by five-color fluorescence of overlapping molecular beacon probes, and one-color fluorescence for the internal control. Results are automatically generated within 2 h and reported as *M.tb*-negative or -positive (with semi-quantification) and RIF sensitive or resistant. The former determination is based on the amplification of any two *rpo* gene regions, and the latter determination is based on a difference of >3.5 amplification cycles of any probe. The Xpert MTB/RIF assay definition files versions 1.0 and 2.0 were used in this study. Data analysis for RIF resistance detection, however, reports results with both the 3.5 and 5.0 cycle threshold differences as per the manufacturer's suggestion.

## Results

### Patient Population and TB Case Classification

**Figure 1:**
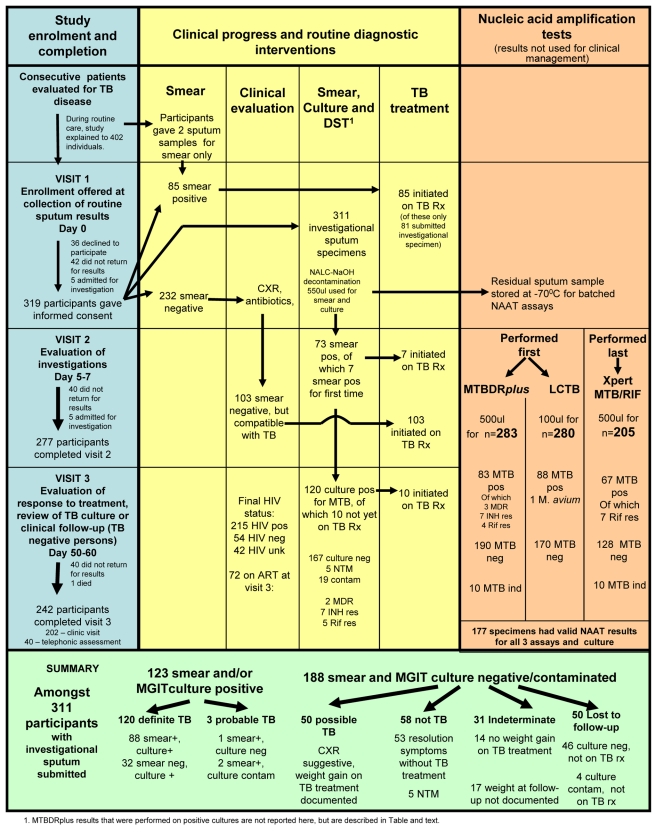
Study algorithm. ART, antiretroviral therapy; contam, contaminated; CXR, chest X-ray; ind, indeterminate; neg, negative; pos, positive; res, resistant; Rx, drug treatment.

During the study period, 402 potential adults with suspected TB presented to the clinic, and 319 agreed to participate (Figure 1). Participants' mean age was 32.4 y (range 19–75 y); 188 (59%) were male (Table 1). Most participants (274, 86%) accepted HIV counseling and testing, among whom 220 (70%) were HIV positive. Eight patients did not provide a sputum sample for study procedures and were excluded from the analysis. Among the 311 patients included in the analysis (Figure 1), 88 (28.2%) were smear- and culture-positive TB cases, 32 (10.2%) had smear-negative, culture-positive TB, and three (0.9%) had smear-positive, culture-negative TB. Culture was contaminated for 19 (6.1%) participants. Among the 188 (60.4%) participants without bacterial confirmation, 50 (26.5%) had possible TB, 58 (30.9%) were classified as not TB (including five patients with NTM), 31 (16.4%) who started TB treatment were classified as indeterminate TB because of failure to gain weight on treatment or because weight at follow-up was not documented, and 50 (26.6%) were classified as indeterminate TB because they were not started on treatment and were lost to follow-up or died.

**Table 1:** Demographics, clinical characteristics, and results of TB diagnostics in 311 adults with suspected pulmonary TB.

Characteristic^a^	All Participants (*n* = 319)	Smear Microscopy, MGIT Culture, and Susceptibility (*n* = 311)	NAAT Performed Directly on Sputum		
			MTBDRplus (*n* = 283)	LCTB (*n* = 280)	Xpert MTB/RIF (*n* = 205)
**Demographics**					
Age in years, mean (range)	32 (19–75)	32 (19–75)	32 (19–57)	32 (19–57)	32 (19–56)
Male gender, number (%)	188 (59)	185 (59%)	165 (58)	161 (58)	115 (56)
**Clinical signs and symptoms at presentation**					
Duration of cough in weeks, mean (range)	4.1 (0–12)	4 (0–12)	3.9 (0–12)	4.0 (0–12)	3.9 (0–12)
Night sweats	287 (90)	281 (90)	256 (90)	253 (90)	186 (90)
Loss of weight	314 (98)	307 (98)	278 (98)	275 (98)	202 (99)
Chest pain	314 (98)	307 (98)	278 (98)	275 (98)	201 (98)
Concurrent extrapulmonary TB symptoms	31 (10)	30 (10)	26 (9)	26 (9)	25 (12)
Pyrexial at presentation	53 (17)	52 (17)	45 (16)	47 (17)	37 (18)
**HIV-related information**					
Agreed to HIV testing	274 (86)	269 (86)	244 (86)	243 (86)	175 (85)
Tested positive	220 (70)	215 (69)	197 (70)	195 (70)	143 (70)
Tested positive: on ART at presentation	17 (5)	17 (5)	12 (4)	11 (4)	8 (4)
Tested positive: mean CD4 count, cells/µl (*n*, range)	214 (166, 0–818)	217 (162, 0–818)	215 (151, 0–818)	214 (149, 0–818)	221 (109, 0–818)
Refused testing	43 (14)	42 (13)	38 (14)	36 (13)	30 (15)
Tested negative	54 (17)	54 (17)	47 (17)	48 (17)	32 (16)
**Bacteriological classification** b					
Smear- and culture-positive	88 (28)	88 (28)	82 (29)	81 (29)	54 (26)
Smear-negative, culture-positive	32 (10)	32 (10)	28 (10)	28 (10)	19 (9)
Smear-negative, culture-negative	166 (52)	166 (53)	150 (53)	148 (53)	115 (56)
Smear-negative, culture contaminated	17 (5)	17 (5)	16 (6)	16 (6)	11 (5)
**Clinical classification**					
Definite TB	120 (38)	120 (39)	110 (39)	109 (39)	73 (36)
Probable TB	4 (1)	3 (1)	2 (1)	2 (1)	1 (1)
Possible TB	51 (16)	50 (16)	45 (16)	44 (16)	40 (20)
No TB	57 (17)	58 (19)	50 (18)	48 (17)	37 (18)
Indeterminate TB status (on TB drugs)	31 (10)	30 (10)	29 (10)	29 (10)	22 (11)
Lost to follow-up, not on TB drugs	56 (18)	50 (16)	47 (17)	48 (17)	32 (15)
***M.tb*** ** case detection**					
Percent with indeterminate results^c^, number/total (percent)	NA	19/311 (6.1)	10/283 (3.5)	0/280 (0)	12/205 (5.9)
Percent positive among those with valid results, number/total (percent)	NA	120/292 (41)	83/273 (30)	88/280 (31)	67/195 (34)
**Detection of RIF and/or INH resistance** d					
RIF resistance, number/total done (percent)	NA	5/89 (6)	8/273 (3)	NA	7/195 (4)
INH resistance, number/total done (percent)	NA	7/89 (8)	10/273 (4)	NA	NA
MDR (INH+RIF resistance), number/total done (percent)	NA	2/89 (1.0)	3/273 (1)^e^	NA	NA

a Values are number (percent) unless otherwise indicated.

b One case was smear-positive, culture-negative, and two cases were smear-positive, culture contaminated.

c No indeterminate smear results; for MGIT culture, indeterminate = contaminated; for Xpert MTB/RIF, indeterminate = error or other result.

d MGIT susceptibility testing done on selected isolates including all cultures where NAAT tests detected resistance.

e Two cases were culture-positive with phenotypic-confirmed MDR; a third case was culture-negative.

NA, not applicable.

### Case Detection by NAAT Assay

Sufficient sputum sample was available to perform NAAT analysis using Xpert MTB/RIF in 205 (64%) participants, MTBDRplus in 283 (89%) participants, and LCTB assay in 280 (88%) participants. There was no significant difference in mean age, gender, smear microscopy, culture, and HIV status between patients in whom the different NAAT assays were performed (all comparative *p*-values>0.05). Overall, NAAT analysis yielded a positive result for *M.tb* in 33% (67/205) by Xpert MTB/RIF, 29% (83/283) by MTBDRplus, and 31% (88/280) by the LCTB assay. Among smear-negative participants (*n* = 227), the proportion of NAAT tests yielding a positive result for *M.tb* was 11.8% (17/143), 6.7% (13/194), and 6.1% (12/199) for Xpert MTB/RIF, MTBDRplus, and LCTB, respectively.

Amongst the NAAT tests, the highest rate of indeterminate or invalid test results was observed for Xpert MTB/RIF (12/205, 5.9%) due to power failures during instrument performance before an uninterrupted power supply was installed (*n* = 2), inability to determine presence or absence of *M.tb* due to improper sample processing (cartridge error) or PCR inhibition (reported as “invalid results”) (*n* = 5), probe check failure (reported as “error”) (*n* = 4), and operator error (*n* = 1). Of these invalid results there was sufficient residual material to re-analyze seven samples, which were then included in the sensitivity and specificity calculations. Only 2.3% of MTBDRplus assays were indeterminate (due to positive *M.tb* control [TUB] band detection issues). None of the LCTB tests results were indeterminate.

### NAAT Sensitivity and Specificity

As detailed in Table 2, compared to MGIT culture, the lowest sensitivity was observed for smear microscopy (59%, 95% CI 47%–71%), followed by MTBDRplus and LCTB with identical performance (76%, 95% CI 64%–85%), and Xpert MTB/RIF (86%, 95% CI 76%–93%), with the highest sensitivity. Sensitivity estimates did not differ for each NAAT when test results were included for specimens not having been tested on all NAAT formats. These results were as follows: smear microscopy, *n* = 289, sensitivity 59% (95% CI 49%–68%); MTBDRplus, *n* = 254, sensitivity 74% (95% CI 64%–81%); LCTB, *n* = 236, sensitivity 75% (95% CI 67%–84%); Xpert MTB/RIF, *n* = 182, sensitivity 86% (95% CI 76%–93%). Specificity was 100% for smear microscopy and >96% for all three NAAT assays. Among culture-negative TB cases, clinical classifications for participants with positive NAAT results were as follows: Xpert MTB/RIF, possible TB (*n* = 1), not TB (*n* = 1), and indeterminate TB status (*n* = 1); MTBDRplus, indeterminate TB status (*n* = 3); and LCTB, indeterminate TB status (*n* = 2).

**Table 2:** Test performance (including comparison to clinical case definitions) for smear microscopy, MGIT culture, MTBDRplus directly on sputum, LCTB, and Xpert MTB/RIF assays stratified by smear microscopy and HIV status.

Test Performance Measure^a^	Smear Microscopy	MGIT Culture	NAAT Performed Directly on Sputum		
			MDRTBplus	LCTB	Xpert MTB/RIF
**Comparison to MGIT culture (** ***n*** ** = 177)**					
Sensitivity	59 (47–71)	NA	76 (64–85)	76 (64–85)	86 (76–93)
Specificity	100 (96–100)		97 (92–99)	98 (93–99)	97 (92–99)
PPV	100 (91–100)		94 (84–98)	92 (87–99)	95 (86–99)
NPV	80 (72–86)		87 (79–92)	87 (79–92)	92 (85–96)
**Comparison to MGIT culture (HIV-positive cohort only, ** ***n*** ** = 124)**					
Sensitivity	54 (38–69)	NA	70 (54–83)	70 (54–83)	84 (69–93)
Specificity	100 (95–100)		96 (89–99)	98 (93–100)	96 (89–99)
PPV	100 (85–100)		91 (76–98)	97 (83–100)	92 (79–98)
NPV	80 (70–87)		85 (76–92)	86 (77–92)	92 (84–97)
**Comparison to MGIT culture (HIV-negative cohort only, ** ***n*** ** = 26)**					
Sensitivity	66 (35–90)		75 (43–95)	75 (42–94)	83 (52–98)
Specificity	100 (70–100)		100 (76–100)	100 (76–100)	100 (76–100)
PPV	100 (63–100)		100 (66–100)	100 (66–100)	100 (69–100)
NPV	79 (52–93)		82 (56–96)	82 (56–96)	88 (62–98)
**Comparison to clinical case definition “Any TB including definite, probable, and possible TB” (** ***n*** ** = 177)**					
Sensitivity	40 (30–50)	66 (56–75)	51 (40–60)	51 (40–60)	58 (48–68)
Specificity	100 (95–100)	100 (95–100)	96 (88–99)	97 (91–99)	97 (91–99)
PPV	100 (91–100)	100 (94–100)	94 (84–98)	96 (87–99)	97 (88–99)
NPV	56 (47–64)	69 (59–77)	59 (50–68)	60 (51–68)	63 (54–72)
**Percent detection** b					
Smear-positive, culture-positive^c^, number/total (percent)	40/49 (81)	49/49 (100)	46/49 (94)	47/49 (96)	47/49 (96)
Smear-negative, culture-positive^d^, number/total (percent)	0/18 (0)	18/18 (100)	5/18 (28)	4/18 (22)	11/18 (61)
Smear-negative, culture-negative, number/total (percent)	0/107 (0)	0/107 (0)	3/107 (3)	1/107 (1)	3/107 (3)

All tests performed on the same 177 sputum specimens. Confidence intervals 95%.

a All values are percent (95% CI) unless otherwise indicated.

HIV status distribution was as follows: HIV-positive, 124; HIV-negative 26; HIV status unknown, 27.

b Amongst 177 cases where all tests were done, 49 were smear-positive, culture-positive; 18 were smear-negative, culture-positive; 107 were smear-negative, culture-negative; in three cases NTM was isolated.

c Where any of the three smears taken during the study period were positive.

d Where all of the three smears taken during the study period were negative.

NAAT test performance amongst the cohort of HIV-uninfected participants had similar sensitivities to test performance on the entire cohort, although the confidence intervals were wide on account of the small numbers. However, amongst HIV-infected participants MTBDRplus and LCTB sensitivities dropped, while that of Xpert MTB/RIF assay remained similar to that of test performance in the entire cohort. As expected for all three NAAT assays, sensitivity was higher among smear-positive than among smear-negative patients (Table 2). Amongst smear-negative, culture-positive cases, Xpert MTB/RIF had the highest sensitivity, 61%, detecting 11/18 cases.

The sensitivity for diagnosis of any TB (smear- and/or culture-positive TB plus possible TB), was 40%, 66%, 51%, 51%, and 58% for smear, culture, MTBDRplus, LCTB, and Xpert MTB/RIF, respectively.

### Detection of Drug Resistance by NAAT

**Figure 2:**
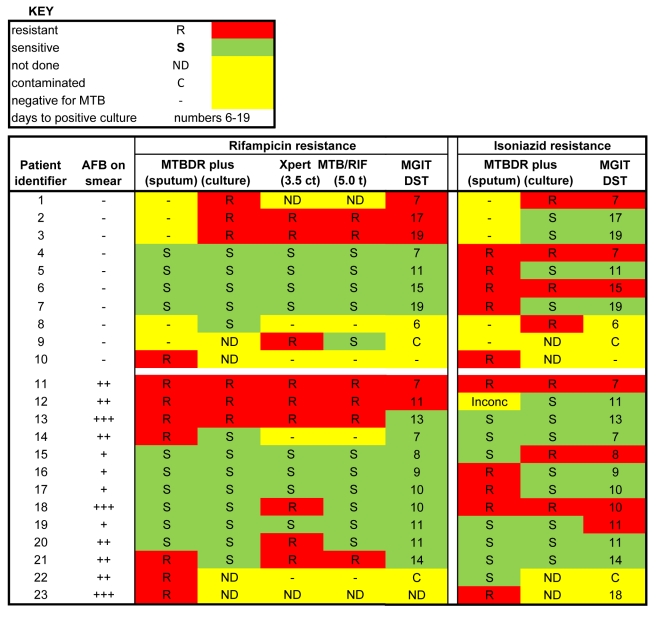
Heat map showing drug susceptibility profiles from 23 samples based on Xpert MTB/RIF, MTBDRplus on sputum, MTBDRplus on cultured isolates, and phenotypic culture (MGIT DST). The 23 samples were from a cohort of 311 participants. The heat map shows samples represented in rows and assigned numerical patient identifiers and testing methodologies in columns. Three colors are used to indicate the results: red, resistant; green, sensitive; yellow, not done, negative for *M.tb*, contaminated, or inconclusive. The samples are sorted into AFB-negative or -positive, with RIF and INH profiles in blocks side by side. Two columns are shown for the RIF results generated from the Xpert MTB/RIF using the amplification cycle threshold maximums 3.5 and 5.0. MDR TB was identified in two patients.

Phenotypic DST results were available for 89 participants, and identified two MDR strains, five INH mono-resistant strains, and three RIF mono-resistant strains. Resistance was detected by MTBDRplus (on sputum or culture) and/or Xpert MTB/RIF in 23 patients (Figure 2). Xpert MTB/RIF identified RIF resistance in nine patients (using the amplification cycle threshold maximum 3.5 of Xpert MTB/RIF software version 1), of which three were not reported as RIF-resistant by other DST methods. These are likely false-positive RIF resistance results, as these samples were reported as RIF-sensitive by Xpert MTB/RIF when using a maximum 5.0 amplification cycle threshold (as per Xpert MTB/RIF software versions 2 and 3). The MTBDRplus test directly on sputum identified eight patients with RIF-resistant TB, seven of these had AFB-smear-positive TB. Two were confirmed by MGIT DST, three were sensitive by MGIT DST, one was culture-negative, one culture was not done, and one culture was contaminated. MTBDRplus directly on sputum did not identify three smear-negative isolates with RIF resistance on phenotypic MGIT DST. MTBDRplus performed on culture isolates identified six patients with RIF resistance, of which five were confirmed by MGIT DST.

INH resistance was detected in ten patients using the MTBDRplus test directly on sputum. Of these, four were also resistant on MGIT DST and MTBDRplus on cultured isolates, four were INH-sensitive by MGIT DST and MTBDRplus done on cultured isolates, while one was negative for *M.tb* on MGIT. MTBDRplus directly on sputum missed INH resistance identified by MGIT DST in two cases, one of which was AFB-smear-negative.

## Discussion

This is a real-world comparison of different TB sputum detection technologies, integrated within a national TB screening guideline. The sensitivity of a single NAAT test compared to a single MGIT culture in our cohort of South African outpatients with suspected pulmonary TB (70% HIV-co-infected) was higher for Xpert MTB/RIF 86% (76%–93%) than MTBDRplus 76% (64%–85%) and LCTB 76% (64%–85%). This difference in sensitivities was especially prominent for the diagnosis of pulmonary TB in HIV-infected individuals (84% versus 70% and 70%, respectively) and among smear-negative, culture-positive patients (61% versus 28% and 22%, respectively). The potential underpowering of this limited sample size should be noted, and it should be clarified that the confidence intervals for all three NAATs do overlap (even with the sample size increased to 289 by including samples not tested by all assays); however, there is no confidence interval overlap between the Xpert MTB/RIF assay (76%–93%) and smear microscopy (47%–71%). This therefore supports the WHO policy that the Xpert MTB/RIF should be the initial test in adults with HIV infection suspected of having TB and can replace smear microscopy. The sensitivity of a single Xpert MTB/RIF assay in our Johannesburg cohort was slightly lower than in the landmark multi-country study (86% versus 92.2% overall and 61% versus 72% for smear-negative, culture-positive specimens) by Boehme et al. [16]. This may be due to differences in study population, as HIV infection rates reported in the multi-country study ranged from 1.7% to 76% across sites [16]. Although the two South African sites (Durban and Cape Town) involved in the multi-center Xpert MTB/RIF study [16] reported HIV infection rates (71.4% and 76.1%, respectively) similar to that found in our Johannesburg population (70% HIV-infected), breakdown of a single Xpert MTB/RIF test compared to a single culture result from these sites was not provided. A recent study [22] performed in a West African (Tanzania) population using a single Xpert MTB/RIF test reported sensitivities of 84.1% overall and 61% for smear-negative, culture-positive isolates, similar to the results in our study. A more recent study also from South Africa in the Cape Town population reports even lower sensitivities of 78.1% overall (performed on raw or processed sputum stored at −20°C) and 55% for smear-negative, culture-positive samples (1 ml unprocessed archived sputum) [23]. This latter study further reported a sensitivity of the Xpert MTB/RIF assay among HIV-infected individuals of 69.6% (*n* = 46), which, although lower than our study findings (84%, *n* = 124), was not significantly different (*p* = 0.09) from the sensitivity reported for the HIV-uninfected group in their study (82.9%, *n* = 82) [23]. All together, these studies and our findings provide evidence of the much improved performance of the Xpert MTB/RIF test compared to smear microscopy. Our findings further show the superior sensitivity of the Xpert MTB/RIF compared to the MTBDRplus and the LCTB assays, especially in the context of HIV co-infection. Some studies have reported Xpert MTB/RIF performance compared to other NAATs not evaluated in our study: the sensitivity of the Xpert MTB/RIF is reported to be higher than that of COBAS Amplicor MTB (Roche) (94% versus 86.8%) and similar to that of ProbeTec ET MTB Complex Direct Detection Assay (BD) (83.7% versus 83.9%) [16]; the sensitivity of the Xpert MTB/RIF assay is reported to be 79%, compared to an in-house IS*6110*-TaqMan real-time PCR assay with 84% sensitivity [24]; the Xpert MTB/RIF is suggested to be as good as the Gen-Probe MTB (Gen-Probe), but no data are available [25].

In our study, the decreased sensitivities of all tests (smear, culture, and NAATs) when using “clinical TB” as a gold standard instead of MGIT culture reflect the paucibacillary nature of pulmonary TB in a community of high HIV seroprevalence and the preference of clinicians to potentially overtreat than undertreat TB in HIV-infected individuals. Amongst these cases, confirmation of TB could be improved through additional MGIT cultures or additional Xpert MTB/RIF assays [16]. However, we elected to remain with this study design (one specimen sample for all investigational NAATs and MGIT culture) as it more closely resembles current South African National TB Control Programme guidelines, and may remain practicable should Xpert MTB/RIF be implemented into routine diagnostic algorithms.

We further compared the assays' performances for the diagnosis of drug-resistant TB. Xpert MTB/RIF can detect mutations in the *rpoB* gene which occur in 95%–99% of RIF-resistant isolates [26]–[29] and are considered a good indicator for MDR TB [30]. The MTBDRplus assay is able to detect *katG* and *inhA* gene mutations that confer INH resistance in phenotypically resistant INH isolates, in addition to *rpoB* gene mutations. The LCTB assay does not detect mutations in resistance-determining regions of *M.tb*. Regarding RIF resistance, over-reporting has previously been described for the Xpert MTB/RIF assay compared with phenotypic DST [16]. Boehme et al. [16] further investigated isolates reported by Xpert MTB/RIF as RIF-resistant, and established by gene sequencing the presence of resistance-associated *rpoB* mutations or mixed infection with wild-type and mutant strains in the same culture. We did not genotype our resistant isolates further but initially observed a higher yield of the Xpert MTB/RIF assay for diagnosis of RIF resistance compared to MTBDRplus or MGIT DST. On re-evaluation using the new recommended software amplification cycle threshold of maximum 5.0, no discrepancies with MTBDRplus were found. We also observed a loss of detection of RIF and INH resistance between MTBDRplus directly on sputum and MTBDRplus on culture isolate. This difference could be due to the presence of a mixed-drug-susceptible and drug-resistant population with different growth potentials [31].

Overall, of 23 resistant samples detected by any methodology amongst 311 patients, we found nine discrepancies between phenotypic and genotypic results. In practice, discrepancies may lead to inappropriate management of TB, with unnecessary exposure to potentially toxic drugs or suboptimal treatment; however, the small sample size limits the full powering for DST accuracy testing.

In addition to the investigation of the Xpert MTB/RIF and MTBDRplus NAAT tests, this study also investigates the new LCTB NAAT assay, which may find place in laboratory settings for cost-effective high-throughput rapid screening (76% sensitive) in place of smear microscopy (59% sensitivity), with similar turnaround times.

A limitation of our study is that NAAT assays were performed on frozen aliquots, while smear microscopy and MGIT culture were performed on fresh samples. This may have impaired *M.tb* detection, and reduced the sensitivity of the NAATs in comparison to culture. In addition, the resuspension of the single processed sputum in ∼2 ml of buffer, as opposed to the recommended 1.5 ml, increased the sample volume, resulting in a dilution and possibly reduced NAAT sensitivities. Freezing of sample aliquots may have caused bacterial disintegration, and consequent suboptimal performance of Xpert MTB/RIF, which relies on capturing whole (intact) bacteria. Several other studies too have recently reported Xpert MTB/RIF assay performance using stored samples: 217 samples from three sites within the western United States processed by NALC-NaOH and then stored at −80°C showed sensitivities of 98% for smear-positive and 72% for smear-negative samples [25]; 125 smear-negative clinical specimens processed by NALC-NaOH and then stored at −80°C for up to 10 y had reported sensitivities (on 1 ml) of 75.3% on the Xpert MTB/RIF assay [32]; 97 clinical specimens processed by NALC-NaOH and then stored at −80°C before Xpert MTB/RIF testing had reported sensitivities of 79% [24]; and the Cape Town study also tested the Xpert MTB/RIF assay using archived specimens, as mentioned above [23]. Despite these limitations, Xpert MTB/RIF still showed superior performance among all NAATs. In favor of the Xpert MTB/RIF assay design is the sample input volume of processed sputum of 500 µl, compared to 100 µl used for the LCTB assay, and product detection using automated, more sensitive fluorescence, not visual detection as with the MTBDRplus assay. Although the Xpert MTB/RIF assay invalid rate appeared higher than previously documented, the use of an uninterrupted power supply did improve result reporting, and should therefore be considered during field implementation.

It has been estimated that the diagnosis of active TB with a sputum-based assay with a sensitivity of 85% and specificity of 97% has the potential to save more than 400,000 lives per year [33]. The only NAAT assay that achieved these targets in our study was Xpert MTB/RIF. Combined with the fast turnaround time and the potential for point-of-care implementation (latter not evaluated in this study), the assay could revolutionize TB diagnosis. Already in a first implementation study of the Xpert MTB/RIF assay [34] in sites in South Africa, Peru, and India, and totaling 6,648 participants, use of the Xpert MTB/RIF assay reduced the median treatment duration for smear-negative TB from 56 d to 5 d. Further research is needed to determine how best to integrate this assay into current TB diagnostic algorithms and to improve our understanding of the prevalence and causes of discrepant drug resistance profiles.

The implementation of point-of-care testing including NAATs such as the Xpert MTB/RIF will need to be assessed for appropriate management of quality assurance, the adequacy of clinic resources (infrastructural and human), data collection, acceptance by patients and health care providers, and affordability, especially in resource-constrained settings.

## Abbreviations

AFB: acid fast bacilli
DST: drug sensitivity testing
INH: isoniazid
LCTB: LightCycler Mycobacterium Detection
MDR: multidrug-resistant
MGIT: Mycobacteria Growth Indicator Tube
*M.tb*: *Mycobacterium tuberculosis*
NAAT: nucleic acid amplification technology
NALC: N-acetyl-L-cysteine-sodium hydroxide
NTM: non-tuberculous mycobacteria
RIF: rifampicin
TB: tuberculosis
WHO: World Health Organization
